# The influence of APOEε4 on cognition in Hispanics: The Panama Aging Research Initiative ‐ Health Disparities Study

**DOI:** 10.1002/alz70856_101032

**Published:** 2025-12-24

**Authors:** Alcibiades E Villarreal, Giselle Rangel, Adam E. Tratner, Diana Carolina Oviedo, Maria B Carreira, Sid E. O'Bryant, Estefani C. Sanchez, Carolina Rodriguez, Josefina Fletcher, Anna Victoria Díaz Ramírez, Xenia E. Arosemena‐Aguero, Pahola Araujo, Ricardo Williams de Roux, Rainier Rodríguez Balaguer, Marilisa Díaz, Nelson Novarro‐Escudero, David Alberto Anguizola Tamayo, Lizeth Pinilla Aguilar, Julio Villarreal, Judy Mendoza, Verónica Billingslea, Jemila Juárez, Karen Courville, Sassil Zuridaday Pitti Degracia, Héctor Torres, Aron Benzadon, Bruno Hammerschlag, Ariel Saldaña, Lizzetha Yolanis Quinterio Girón, Dámaso Moisés Díaz Solís, Laura Mercedes Cerrud Castillo, David Dondis, Fernando Sucre, Alexis Ariel Palacios Adames, Andrés Eduardo Bernales Crespo, Sofía A Rodriguez, Natalia López, Gabrielle B Britton

**Affiliations:** ^1^ Sistema Nacional de Investigación (SNI), Panamá, Panamá, Panama; ^2^ Instituto de Investigaciones Científicas y Servicios de Alta Tecnología (INDICASAT AIP), Centro de Neurociencias, Panamá, Panamá, Panama; ^3^ Florida State University, Panama, Panama, Panama; ^4^ Escuela de Psicología, Universidad Santa María la Antigua (USMA), Panamá, Panamá, Panama; ^5^ Institute for Translational Research, University of North Texas Health Science Center, Fort Worth, TX, USA; ^6^ Instituto de Investigacion Innovacion y Gestion del Conocimiento, Caja de Seguro Social (CSS), Panama, Panama, Panama; ^7^ Hospital The Panama Clinic, Panamá, Panamá, Panama; ^8^ Hospital Pacífica Salud, Panamá, Panamá, Panama; ^9^ Servicio de Neurología, CIDELAS‐CSS, Panamá, Panamá, Panama; ^10^ Hospital Paitilla, Panamá, Panamá, Panama; ^11^ Hospital Dr. Gustavo Nelson Collado, Chitré, Herrera, Panama; ^12^ Caja de Seguro Social, Panamá, Panamá, Panama; ^13^ Servicio de Radiología, Hospital Paitilla, Panamá, Panamá, Panama

## Abstract

**Background:**

The apolipoprotein E (APOE) gene is a well‐established risk factor for Alzheimer's disease. This study aims to investigate the relationship between APOE genotype and cognitive aging in a sample of Hispanics. We also present data from the research protocol of an ongoing clinical study within the PARI‐HD program. The main objective is to evaluate the diagnostic and prognostic value of biomarkers in blood, cerebrospinal fluid and magnetic resonance imaging for MCI, AD and PD in Panamanian older adults (≥ 65 years of age).

**Method:**

Participants (*n* = 725) were divided into 2 groups, an outpatient cohort (*n* = 269) and a community cohort (*n* = 456) and underwent comprehensive neuropsychological assessments. For the clinical study of biomarkers, we obtained demographic data, functional status and neuropsychological evaluations. Also, CSF, MRI and fasting blood samples were obtained. Participants were divided into: MCI, AD, PD, and without cognitive impairment groups.

**Result:**

APOE analysis shows that the ε4 frequency was 32.3% among the outpatient cohort compared to 23.8% among the community cohort. Results showed that ApoEε4 status was a significant predictor of cognitive impairment. In the outpatient cohort, carrying at least one copy of the APOEε4 allele was significantly associated with worse performance on attention.

**Conclusion:**

These findings suggest that APOEε4 confers risk for cognitive deterioration in Panamanian population, highlighting the importance of considering genetic and demographic factors in understanding the heterogeneity of cognitive impairment. Our study is the first of its kind in Panama and one of few in the LAC region, setting a milestone in exploring CSF protein analysis and MRI techniques associated with MCI, PD and AD in Hispanic/Latino older adults.

